# Commencements

**Published:** 1898-06

**Authors:** 


### COMMENCEMENTS.

### Tennessee Medical College—Dental Department.

The annual commencement exercises of the Dental Department of the Tennessee Medical College were held in Staub's Gpera House, Knoxville, Tenn., on Tuesday evening, March 29, 1898.

The annual address was delivered by Rev. James A. Duncan, D.D., and the charge to the graduates by Dr. J. C. Cawood.

The number of matriculates for the session was fourteen.

The degree of D.D.S. was conferred on the following graduates by Professor J. T. Henderson, A.M., president of the board of trustees: E. M. Caffey, North Carolina; James L. McCarty, Kentucky; R. C. Lowrance, North Carolina; A. Brooks, Tennessee; W. C. Mayr, Kansas. Total 5.

### University of Buffalo.

The seventh annual commencement exercises of the Dental Department of the University of Buffalo were held in connection with those of the Department of Medicine and Pharmacy, at Music Hall, in the city of Buffalo, Tuesday evening, April 26th.

Upon the recommendation of the Faculty and by vote of the Council the Chancellor conferred upon the following students, who had complied with all the requirements, the Degree of Doctor of Dental Surgery:

| NAME | RESIDENCE |
|---|---|
| George Washington Averell | New York |
| Edward Barber | Ontario |
| Edgar Atheling Bartlett | Ontario |
| Charles Wesley Borland | New York |
| William Arthur Bostwick | New York |
| Byron Alfred Brown | New York |
| George Brown | New York |
| Jacob Harry Brown | New York |
| Charles Felix Buckland | New York |
| Harry Dayton Burghardt | New York |
| Egerton Bidwell Cays | Ontario |
| Claude Robert Christopher | New York |
| John Emerson Cole | Ontario |
| Arthur Hurbert Consaul | Ontario |
| Herschel Jacob Cull | New York |
| Harry Washington Day | New York |
| George Martin Decker | New York |
| William Brown Dills | New York |
| Kernon James Donahoe | New York |
| John Lockhart Dudley | Ontario |
| Moss Burton Eshleman | New York |
| Raymond Reece Evans | New York |
| Frank Leslie Frank | New York |
| Katharine Magdelane Graf | New York |
| Allen Lewis Green | New York |
| John Nickles Hanna | New York |
| Albert Devitt Heist | Ontario |
| Harry Collins Howell | Ontario |
| George Washington Jones | New York |
| Albert Hugh Jung | New York |
| Otto Charles Kalbfleisch | Ontario |
| Henry Wilford Kitching | New York |
| Charles Henry Laborn | New York |
| Christian Albert Landel | New York |
| Clarence Edw Lauderdale | New York |
| Clinton Henry McCallum | Ontario |
| Arthur Amzi Mix | New York |
| Herbert Butler Morris | Ontario |
| Robert Murray | Ontario |
| Judson Hedden North | New York |
| George Albert Northup | New York |
| William Wilton Paull | New York |
| John Robert Quigg | New York |
| Harry Budd Randall | Pennsylvania |
| Grace Greenwood Rankin | Ohio |
| Howard Frank Redfield | New York |
| Earnest Emerson Rice | Ontario |
| Samuel Emerson Salisbury | New York |
| Edw Leonard Schlottman | New York |
| William Francis Sheahan | Ontario |
| William Derwood Slacer | New York |
| Miles Montaine Smith | New York |
| Josephine Harriett Spellman | N York |
| Louis Almon Squires, Ph. B. | New York |
| Ella Amanda Staley | New York |
| George Stevenson | Ontario |
| Duncan Stewart | Ontario |
| Eli Jay Sweet | New York |
| Charles Dickinson Tefft | New York |
| John Clar Burroughs Tooke | New York |
| George Carman Van Marter | New York |
| Francis Harold Wallace | Ontario |
| Louis Eidt Wettlaufer | Ontario |
| Jay Leonard Wilson | New York |
| Fred Arthur Woodmansee | Penn'vania |
| William Davison Worrell | New York |

### Chicago College of Dental Surgery.

The sixteenth annual commencement exercises of the Chicago College of Dental Surgery (Dental Department of Lake Forest

University) were held in Central Music Hall, Chicago, Ill., on Wednesday afternoon, April 6, 1898.

The doctorate address was delivered by Truman W. Brophy, M.D., D.D.S., LL.D., and the valedictory by Guy Lawrence Bergen, D.D.S.

The degree of D.D.S. was conferred on the following graduates by Truman W. Brophy, M.D., D.D.S., LL.D., president of the college:

Elmer K Adams

William S Adair

George W Aldrich

Hugh F Armstrong

Squire A Arnold

Frederick Brethour

James A Bell

Fay G Babcock

William H Barricklow

Frank B Bassett

Adelbert C Bauer

Guy L Bergen

Alfred W Berger

Jacob Bloomenthal

King Brooks

Stephen W Brundage

C Arthur Bourn

John P Buckley, Ph G

David C Budge

George B Bunyan

Joseph I Bush

Thomas C Campbell

Thomas F Campbell

Will A Cann

Jonas F Canode

William J Charters

William G Coffey

Ralph E Collins

Oscar H Cole

Martin J Conley

Frederick D G Cook

Robert C Corbus

LeGrand M Cox Jr

George T Cress

Thomas E Crowley

Joseph P Cruise

Robert J Cruise

George W Cunningham

Myron E Curtiss

John W Daniels

Lawrence C Doherty

Joseph T Davies, M D

Frank H Davis

Ira F Dillman

James T Doherty

William M Dolan

Ernest W Draver

George Drury

Hiram E Eells

Elmer W Elliott

Harry V Epperson

John W Epperson

Charles H Evans

Harry L Foster

James D Fair

Jesse Filmar

Christian C Fleischer

James F Flynn

Albert W Fossum

Harry A Foster

Frank Freymann

Vernon M Gregg

Edwin L Garrett

Louis A Gebhardt

George W Geenty

Edward D George

Elmer G Gibson

Walter C Golbeck

Thomas F Gorman

George Gow

George N Green

Carl J Grove, Ph G

Thomas L Grisamore

Oliver H Harden

Andrew J Harris Jr

Norman B Hall

John A Heaton

Carl Heper, Ph G

John N Higgins

Jesse L Hitz

E Everett Holden

Frank J Hoeschler

Julius L Holton

Samuel R Hopkins

George W Humpidge

A C Hutchinson Jr

Charles H Jefferis

Fred L Jenkins

Albert A Jessup

William Kamin

Edwin A Kartach

Oscar M Kenck, M A

Friend B Kenward

Warren S Kern

John W Kleeber

David E Kulp

Frank Kyle

Peter P Lobig

Edward E Lamberton

Leo H Lawson

Noah A Lee

Garrett Lighthart

Carl Lindstrom

James D Locke

Thomas Lockie

Charles J Long Jr

J W H Loppenthein, Ph G

L P M O

Earl G McAbery

John F Maloney

Charlie R Mead

George W C Meyer

Frederick G Miller

Gove L Miller

Frank D Miner

Rudolph A Mittlestadt

William J Montgomery

William E Moore

John A Morrison

Peter A McMillan

Peter C McNiff

Joseph F McParland

William Mullin

Walter L Neil

Ralph E Nelson

Charles W Nerud

Arthur B Oren

Royal H Pierce

Harry B Parrish

Clayton A Patterson

Roscoe B Power

Stephen E Pettit

Orren E Phillips

Robert E Raleigh

Edgar R Riggs

Clarence M Rankine

Charles F Rockey

Ellis M Rogers

Isaac N Rosenbelt

Marx Ruge

William H Sargent

William A Sebolt

Frederick H Shannon

Henry A Shannon

Walter H Shoening

James M Selvis

Benjamin B Sharp

Richard C Smith

Bert L Snashall

Marc A Southworth

Alexander M Sowler

Burt Le R Spellman

Wilford C Stryker, B S

Carl Sholtz

George E Taylor

Joseph H Teter

George W Turner

Joseph J Volker

Howard L Waide

Francis X Walsh

Albert O Watland

William E Webster

Ralph C Williams

Arthur C Willman

Total 161.

### Atlanta Dental College.

The annual commencement exercises of the Atlanta Dental College were held in the Grand Opera House, Atlanta, Ga., on Tuesday evening, March 29, 1898.

Professor William Crenshaw read his report as dean of the college, and the valedictory address was delivered by John C. Richard, Jr., D.D.S.

The number of matriculates for the session was one hundred and eighty-five.

The degree of D.D.S. was conferred on the following graduates by Judge W. R. Hammond:

| NAME. | RESIDENCE. | NAME | RESIDENCE |
|---|---|---|---|
| W B Alford | South Carolina | F C Latham | Florida |
| W W Allen | Georgia | H L Meldrim | Georgia |
| B R Anderson | North Carolina | M M Minter | Georgia |
| J C Anderson | Georgia | H M Moore | Georgia |
| H C Bazemore | Tennessee | Henry Moor | Georgia |
| T E Barker | Alabama | F W McCracken | North Carolina |
| J W Back | Florida | E A McMillan. | South Carolina |
| J M Brannon | Georgia | L E McTyre | Georgia |
| L D Baggs | Georgia | L B McLaurin | Mississippi |
| R L Branyon | South Carolina | N J Naul | Mississippi |
| L A Brewster | Louisiana | L D Owen, M D | Tennessee |
| P E Callihan | North Carolina | J L Pope | Mississippi |
| J G Caldwell | Mississippi | J A Peterson | Georgia |
| P T Dashwood | England | R H Park | Georgia |
| W H Elder | Georgia | C E Pittman | Georgia |
| G Enloe | Florida | W A Park | New York |
| W G Ford | Georgia | J D Regan | North Carolina |
| Theo Foster | Connecticut | S B Raby | Texas |
| H Griffin | Georgia | J C Richard Jr | Florida |
| B E Goolsby | Georgia | G T Sharpton | South Carolina |
| J E Gachet | Alabama | G W Shackelford | Georgia |
| O P Goodman | Georgia | R G Shanks | Alabama |
| R B Hill | Georgia | W T Simmons | Indiana |
| W T Herndon | North Carolina | H E Satterfield | North Carolina |
| I M Hair | South Carolina | H P Teague | South Carolina |
| C B Hall | North Carolina | J B Tiller | Florida |
| R F Ingram | Georgia | H C Terrell | New York |
| T P Jones | Georgia | T A Terrell | Georgia |
| E L Johnston | Mississippi | F C Watson | Louisiana |
| J I Lee | Alabama | J H White | Louisiana |
| P W Lea | Louisiana | | |

Total 61.

### Pennsylvania College of Dental Surgery.

The forty-second annual commencement exercises of the Pennsylvania College of Dental Surgery were held in the Academy of Music, Philadelphia, on Tuesday evening, April 5, 1898.

The address to the graduates was delivered by Professor Henry Leffmann, M.D., D.D.S.

The number of matriculates for the session was three hundred and seventy-six.

The degree of D.D.S. was conferred on the following graduates by I. Minis Hays, M.D., president of the college:

| NAME. | RESIDENCE. | NAME. | RESIDENCE. |
|---|---|---|---|
| R G Ackley | Canada | Clara J. Kretschman | Pennsylvania |
| J W Adams | Illinois | Irva Lu Ella Kuter. | Illinois |
| HaA Anderson | Bahama Islands | Emil Lanz | Switzerland |
| Janett W Angle | New York | J B Lewin, B A | South Africa |
| G B Aumack | New Jersey | F H Lloyd. | Pennsylvania |
| Clifford D Beale | Pennsylvania | G F Logan | Nova Scotia |
| H W Black | Nova Scotia | H W Lyte | Pennsylvania |
| C B Blackman | Connecticut | G C McBride | Pennsylvania |
| Anna Block | Russia | J J McLaughlin | Massachusetts |
| C Carayannapulo | Roumania | Wm H Manning | Pennsylvania |
| W L Carhart | New Jersey | Oscar Maul. | Pennsylvania |
| Chas E Chase | Pennsylvania | Chas Nair | Pennsylvania |
| F D Clark | Connecticut | T L Napton | Montana |
| La Mancha Clark | New York | H C Neale | Pennsylvania |
| V L Clark | Canada | Reinhard W Nell | Pennsylvania |
| G W Coder | Pennsylvania | Jno T Nicholson | Canada |
| S Collins | New York | W H Norris | Pennsylvania |
| S D Cornish | Pennsylvania | W M Nyer | Pennsylvania |
| Anna G Crothers | Maryland | W L Off | Pennsylvania |
| A Van E Davis | Pennsylvania | H I Oliver | New York |
| S A de Waltoff | Pennsylvania | W A Parker | Pennsylvania |
| J H Drummond | Canada | W H Perdue | Canada |
| J Y Duncan | Canada | G A Pineda | South America |
| Chas W Dunlevy | Georgia | Oswaldo Pochet | San Domingo |
| J G Edwards | France | Chas C Rowe | Canada |
| C E Eilenberger | Pennsylvania | Jno T Rugh | Pennsylvania |
| H L Erb. | Pennsylvania | C S Saylor | Pennsylvania |
| J W Freyling | Pennsylvania | S E Schriner | Pennsylvania |
| R E Gallagher | Pennsylvania | Anna Scott | Pennsylvania |
| F P Gleeson | Rhode Island | P L Showers | Pennsylvania |
| H N Gorham | Canada | M G Silvis | Pennsylvania |
| Florence S Green | Colorado | Jos A Smith | Massachusetts |
| C Grenache | Massachusetts | Wm A Smith | Pennsylvania |
| Louise B Grover | Pennsylvania | Gustav Stanetzky | Germany |
| J S Hall | New York | C C Sticker | Pennsylvania |
| W L Harpel | Pennsylvania | E F Stratford | Pennsylvania |
| E R Hart | Canada | Catharine Sweeney | Pennsylvania |
| Wm F Hayes | New Jersey | H W Tate | Pennsylvania |
| W J Henchey | New York | S S Theller | California |
| A F Henson | Pennsylvania | E L Thomas | Missouri |
| E S Hipple | Pennsylvania | W A Thomas | Missouri |
| Henry Hoppman | Pennsylvania | F S Thome | Missouri |
| John Hutchison | Pennsylvania | C H Tillinghast | New York |
| Claude W Johnson | Pennsylvania | G B Varwig | Pennsylvania |
| John E Johnson | Pennsylvania | W Matlack Ward | Pennsylvania |
| Wm C Keen | Pennsylvania | G N F Wardrop | Canada |
| C L Kelleher | Pennsylvania | R B Webb | Pennsylvania |
| Chas O Kennedy | Missouri | H T Wells | Missouri |
| Nettie L Knepp | Penhsylvania | J E Williamson | Pennsylvania |
| L G Koenig | Pennsylvania | C R Wintersteen | Pennsylvania |

Total 190.

### University of Maryland—Department of Dental Surgery.

The annual commencement exercises of the Department of Dental Surgery of the University of Maryland were held in Music

Hall, Baltimore, Md., on Thursday evening, March 31, 1898.

The address to the graduates was delivered by Rev. H. W. Wharton, D.D., and the class oration by Adelard J. Harper, D.D.S.

The number of matriculates for the session was two hundred and seven.

The degree of D.D.S. was conferred on the following graduates by Bernard Carter, Esq., provost of the university:

| NAME. | RESIDENCE. |
|---|---|
| Wm E Anderson | South Carolina |
| Charles E Beall | Virginia |
| Fred H Brown | Vermont |
| Martin Buhtz | Germany |
| E L Burroughs | Maryland |
| Benj F Carpenter | New York |
| Le Roy J Conger | Canada |
| Halla F Chapman | Kansas |
| Franklin E Clark | New York |
| Ernest J Cooke | Panama, S A |
| Claude S Daly | New York |
| Edw B Dawson | New York |
| A van K Deekens | Maryland |
| Clayton C Eyler | Pennsylvania |
| Welty B Fahrney | Virginia |
| L W Farinholt | Virginia |
| Charles E Fording | Ohio |
| H W Freeman | Illinois |
| R T Gallagher | North Carolina |
| O E Gregoire | Rhode Island |
| Joseph B Grom | New Jersey |
| Abr. Grunberg | Roumania |
| E C Hamilton | Virginia |
| G N Hardesty | Virginia |
| F R Harkinson | California |
| Adelard J. Harper | Massachusetts |
| Pleasant S Hays | Ohio |
| C D Holmes, Jr | Texas |
| John H Judd | North Carolina |
| Jesse E Judd | North Carolina |
| Clinton M Koontz | Maryland |
| Chas W Leonard | New Jersey |
| Thomas S Lewis | Pennsylvania |
| Pierre A Michel | Louisiana |
| M B Milner | Georgia |
| Robert F Moor | Pennsylvania |
| Matt McAndrew | Ontario, Can |
| T W McFadden | Pennsylvania |
| Fred'k C McKee | Quebec, Can |
| W H Nichols | New Hampshire |
| W T Palmer | Iowa |
| D Lee Peacock | Georgia |
| D Irvel Pletcher | Ohio |
| Joseph T Pyles | Maryland |
| F W H Quinlan | New York |
| B C Redfearn | North Carolina |
| Montell B Rudd | Virginia |
| Luther T Smith | North Carolina |
| C L Smither | Virginia |
| V D Shaffner | Nova Scotia |
| G A Sprinkel, Jr | Virginia |
| Albert H Stone | New York |
| Earl Z Stafford | Texas |
| R W Taggart | Vermont |
| Charles A Terry | West Virginia |
| Rob't L Thacker | West Virginia |
| Edwin P Tignor | Virginia |
| Geo H Trump | Maryland |
| G M Warrenfels | Maryland |
| Thomas J Walsh | Vermont |
| L A White | Dist of Columbia |
| Ed R Williams | Pennsylvania |
| W E Williams | West Virginia |
| Joseph E Wright | Maryland |

Total 64.

### Philadelphia Dental College.

The thirty-fifth annual commencement exercises of the Philadelphia Dental College were held in the Academy of Music, on Friday evening, April 1, 1898.

The address to the graduates was delivered by Professor L. Greenbaum, M.D., D.D.S., and the valedictory by Wm. P. Devine, D.D.S.

The degree of D.D.S. was conferred on the following graduates:

| NAME | RESIDENCE |
|---|---|
| James H Abbott | Delaware |
| James W Allen | Pennsylvania |
| Henry R Arends | Nebraska |
| J H P Armatage | Canada |
| Louis Back | New York |
| Cassius M Barns | Pennsylvania |
| Stephen S Benson | New York |
| H H Bethel, A.B | Pennsylvania |
| Sidney H Bigler | Pennsylvania |
| R P Buckley | Massachusetts |
| Thomas J Byrne | Pennsylvania |
| William H Cahill | Connecticut |
| J C Campbell | Canada |
| Thomas J Carrier | Connecticut |
| Noemi Chabut | France |
| William C Chinn | Canada |
| Louis K Cleaver | Pennsylvania |
| John A Clifford | Rhode Island |
| John Common | New York |
| V F Cunningham | Canada |
| William P Devine | Vermont |
| Chas H Doering | Oregon |
| J H Donaldson | Pennsylvania |
| Harry G Dunbar | Canada |
| H S Du Vernet | Australia |
| Thomas J Egan | Minnesota |
| Irwin M Eisaman | Pennsylvania |
| Rupert W Elvy | Australia |
| Harry A Irwin | Pennsylvania |
| Frank B Evans | Iowa |
| D G Farrington | New Jersey |
| John P Fellows | Kansas |
| Bertram C Fern | Pennsylvania |
| L W Fischer | California |
| John C Fox | Pennsylvania |
| H O Frederick | Ohio |
| C E Freeman | Maine |
| Harry B Garrison | Maine |
| George E Girard | New York |
| John A Glenzer | New York |
| H M Goodwin | Canada |
| Robert B Green | New York |
| Joseph Grice | England |
| Almon E Hamlin | Connecticut |
| William G Hand | New Jersey |
| Walter S Hart | Connecticut |
| A H Henderson, Jr | Pennsylvania |
| Geo W Hinkson | Pennsylvania |
| John P Hines | California |
| Harry A Hurd | Iowa |
| W B Hutchens | New York |
| Richard Jeffries | Pennsylvania |
| Miles L Johnson | Pennsylvania |
| M Joseph, D.E.D.P. | France |
| John D Kelly | Pennsylvania |
| Sydney Kerr | Canada |
| Lottie M Kidder | Massachusetts |
| Amalie Klonower | Germany |
| L E Knobeloch | South Carolina |
| George C Knorr | Pennsylvania |
| A M Lafayette | Canada |
| W E F Landers, Jr | Connecticut |
| James E Lang | California |
| Gaylord Lansdowne | Missouri |
| A Van H. Lewis | Pennsylvania |
| P D Luxemburger | Pennsylvania |
| Sarah J Mace | New York |
| A H MacTaggart | Australia |
| J M Mackie | Scotland |
| W W McClarin | Pennsylvania |
| R H McCready | Maine |

| NAME | RESIDENCE |
|---|---|
| W L Birmingham | Pennsylvania |
| Will A Blackburn | Texas |
| Frank L Booth | New York |
| Shirley W Bowles | New York |
| Sylvan Brautman | Roumania |
| Wm F Browning | California |
| Claude Brown | Canada |
| Henry S Brown | Canada |
| Wallace W Bruce | Canada |
| E D McLe d | Connecticut |
| H J McNaughton | Canada |
| W L McWilliams | Pennsylvania |
| Roy L Magoon | Minnesota |
| George L Markle | Pennsylvania |
| Geo A Marshall | Oregon |
| Lloyd L Means | Pennsylvania |
| Jos P Monahan | Massachusetts |
| Charles S Moon | New York |
| Fred J Moon | New York |
| Alfred C Nathan | Australia |
| John J Needham | Pennsylvania |
| Gleni C Newcom | Pennsylvania |
| L G Nickerson | Pennsylvania |
| Alvarez Nugent | Canada |
| Fred R Ogden | California |
| Frank C Parsons | Connecticut |
| Alex Paterson | Australia |
| Jno W Pinkham | Pennsylvania |
| R B Pollock, Jr., M.D., Ph.G | Pennsylvania |
| C W Powers | Canada |
| C O Regener | New York |
| John A Reidy | Pennsylvania |
| Edw S Roberts | North Carolina |
| Walter F Rogers | Pennsylvania |
| V M Rundle | New Jersey |
| Harry N Sackett | New York |
| Frank Sanbern | New York |
| Albert E Seal | Rhode Island |
| M I S Schenck | Pennsylvania |
| W G Seeley | Pennsylvania |
| Frank W Senior | Massachusetts |
| William N Short | Australia |
| Wm C Showalter | Pennsylvania |
| C G Skinner | California |
| Carlos E Smith | Pennsylvania |
| Edw D Sparke | Australia |
| Moore Stevens | Pennsylvania |
| Jos V St Martain | Louisiana |
| Stanton A Stuart | Ohio |
| E E Sullivan | Massachusetts |
| Charles E Swain | Connecticut |
| J Edward Tether | New York |
| Wm H Thomas | Maine |
| S W Thornbury | Pennsylvania |
| John H Torrance | Massachusetts |
| A K Tuttle, A.B | Pennsylvania |
| D R Van Amringe | California |
| R O Van Deusen | New York |
| A N Van Dyke, Ph.G | Pennsylvania |
| Chas H Van Kirk | New York |
| John B Vedder | Oregon |
| A L Westcott | New Jersey |
| William A White | Pennsylvania |
| Geo B Whitten | Maine |
| Charles O Wilhin | Pennsylvania |
| F H Williams | New York |
| George W Wise | Pennsylvania |
| W H Witham | Maine |
| Geo S Writer, Jr | New York |
| Arthur Zentle | Roumania |

Total 141.

### Baltimore College of Dental Surgery.

The fifty-eighth annual commencement exercises of the Baltimore College of Dental Surgery were held in Ford's Opera House, Baltimore, Md., on Friday afternoon, April 1, 1898.

The annual oration was delivered by L. C. F. Hugo, D.D.S., and the valedictory by E. W. Gaynor, B.S., D.D.S.

The degree of D.D.S. was conferred on the following graduates by Professor M. W. Foster, dean of the college:

| NAME | RESIDENCE | NAME | RESIDENCE |
|---|---|---|---|
| R T Allen, B.A | North Carolina | C E Luiz | Ohio |
| H B Anderson | Canada | C B McElrath | West Virginia |
| J E W Armstrong | Pennsylvania | M H McElroy | Rhode Island |
| W F Baker | Pennsylvania | A R McLaughlin | North Carolina |
| W N Barnes | New York | C H Merritt | Maine |
| H N Bernard | New York | H G Morgan | Ohio |
| A C Brewer | Pennsylvania | J H Morrison | Ohio |
| H R Brightbill | Pennsylvania | C R Morton | Nova Scotia |
| E T Byrne | Pennsylvania | P Nixon | Alabama |
| C P Calloway | West Virginia | H D Osborne | Georgia |
| A Chesterfield | Canada | T O Palmer | New York |
| D C Clark | West Virginia | M E Parsons | Maryland |
| G Collins | Maine | M L Perez | Cuba |
| J C Conover | New Jersey | E A Poe | Texas |
| F del Valle | Porto Rico | E R Rhein | Pennsylvania |
| C W Duttman | West Virginia | C R Riddick | North Carolina |
| W Doebbelin | Germany | W C Richardson | Louisiana |
| W O Dunning | New York | T J Ritter | Texas |
| O W Elzey | Maryland | V A Roach | Nova Scotia |
| C V Emelio | Portugal | C M Ryan | New York |
| S Emmich | Mississippi | G E Seay | Virginia |
| J P Finn | Connecticut | R M Sentz | West Virginia |
| W B Fletcher | West Virginia | M S Shoemaker | Pennsylvania |
| E W Gaynor, B.S | Massachusetts | B P Simpson | Massachusetts |
| B J Gorman | Maryland | H E Smith | Pennsylvania |
| F P Gromley | Rhode Island | C W Straight | New York |
| J K Grishman | Tennessee | A P Sylvia | Portugal |
| G Hain | Pennsylvania | P W Tankard | Virginia |
| G F Herring | North Carolina | W O Throop | New York |
| G A Hitch | Delaware | W L Thwaits | New York |
| W G Huntley, B.S | Vermont | C H Tice | New York |
| C E Johnson | Virginia | O J Toujan | Louisiana |
| J M Kelly | New York | D L Towberman | Virginia |
| R E Koehler | Texas | W Wade | Maryland |
| F A Lefurgey | P E Island | O E Wall | Honolulu |
| H M Lever | Canada | C A Wood | Pennsylvania |
| G Loeffler | Maryland | W Wolf, M.D | Maryland |

Total 74.

### Western Dental College.

The eighth annual commencement exercises of the Western Dental College were held in the Coates Opera House, Kansas City, Mo., on Saturday evening, April 2, 1898.

The annual address was delivered by Rev. J. M. Cromer, and the faculty address by Dr. T. H. Cunningham.

The degree of D.D.S. was conferred on the following graduates by Dr. D. J. McMillen, dean of the faculty:

| NAME | RESIDENCE | NAME | RESIDENCE |
|---|---|---|---|
| M L Beason | Missouri | S Locke | New Mexico |
| C P Becker | Virginia | Edwin Marshall | Missouri |
| Augusta H Brady | Sweden | R F McCreery | Missouri |
| Harry M Cannon | Missouri | C W Middleton | Missouri |
| F G Chamblin | Missouri | F B Miller | Missouri |
| G C Cleveland | Illinois | R M Minter | Missouri |
| C L Cleveland | Illinois | J D Moore | Kansas |
| T F Clifford | Missouri | W G Owen | Kansas |
| Levi Cook | Missouri | R S Parsons | Missouri |
| Emmett Craig | Missouri | Ernest Peterson | Kansas |
| E P Dameron | Missouri | J H Rhodes | Kansas |
| L C Frazier | Missouri | T H Richmond | Missouri |
| G A Gruebbel | Missouri | F S Robison | Missouri |
| C J Hamilton | Iowa | G R Rose | Missouri |
| R W Hammons | Idaho | G L Rosier | Missouri |
| Glea Harvey | Missouri | Herman Schneider | Kansas |
| W C Hensley | Wyoming | Chas Skinner | Kansas |
| W E Huffman | Missouri | F R Souders | Kansas |
| S W Hurlburt | Kansas | Harry Stinson | Missouri |
| C R Hubbell | Missouri | Henry Toelle | Missouri |
| F B Jahr | Missouri | H D Wherritt | Missouri |
| T W Keel | Illinois | H P White | Iowa |
| T W Kelton | Missouri | H G Whittlesey | Kansas |
| W B Kenney | Missouri | F B Wiesner | Kansas |
| R C Korff | Missouri | A J Williams | Illinois |
| R L Krueger | Missouri | E B Winslow | Missouri |
| E A Litchfield | Missouri | | |

Total 53.

### Indiana Dental College.

The Nineteenth Annual Commencement Exercises of the Indiana Dental College occurred at English's Opera House, Indianapolis, Tuesday evening, April 12, 1898. The address of the evening was by Dr. Joseph W. Matthews, Dean of the Kentucky School of Medicine, at Louisville. Following is the list of graduates:

| | | |
|---|---|---|
| Horace Lawson Baldwin | Albert Glick | John Wallace McMillan |
| Horace C Beckes | George Rolland Gunder | Robert Clay Mayhall |
| Harry Wolfe Bowers | Arthur Grimes | Clarence Elliott Marsh |
| Harry Craig Carr | Ellsworth Glenn | Joseph L Masters |
| Vere M Chappell | E Allen Hodges | Everette Leroy Murphy |
| Rose Marian Cooper | Samuel George Hoblit | Owen L Mather |
| Ray Frank Coddington | Wilber K Harlan | W Frank Purnell |
| Patrick Francis Campbell | Charles Leslie Hallam | Arnet Earl Ross |
| Clarence Wilbur Dicks | Joseph Vincent Ireland | Orton B Smith |
| James Lenard Dillon | Charles Roland Jackson | Fred D Sparks |
| Charles L Davis | Albert Lee Jennings | HarryBromwell Summers |
| James H Dean | Harry Elmor Jackson | William Grant Tull |
| William Arthur Fox | Charles F Kraning | Paul Edwin Vize |
| J Wesley Fry | George Lawrence Lane | John Robinson White |
| Everett B Fewell | Frank S Martin | Williams Edw Walker |
| Louis Parker George | Edgar D McLaughlin | Henry Albert Wahl |
| William D Graham | Arthur Alman Metsker | |
| Malcolm Thos Goodman | Bennett Frank'n Michael | |

Total 52.

### Columbian Dental College.

The annual commencement exercises of the Columbian Dental College were held at Handel Hall, Chicago, Ill., on Tuesday afternoon, April 5, 1898.

The annual address was delivered by Rev. J. W. Fifield, and the valedictory by Louis C. Neeb, D.D.S.

The degree of D.D.S. was conferred on the following graduates by George T. Carpenter, M.D., D.D.S., president of the college:

| | | |
|---|---|---|
| Arthur J Anderson | D Lawrence Frazee | Charles A Sultze |
| Benjamin J Baskerville | Lewis E Fisher | Louis C Wagner |
| Omar H Banta | Edward V Harraman | Munson L Ward |
| Grant Craig | Ambrose J Knetzger | L Linville Whitt |
| Richard B Davenport | Louis C Neeb | |

Total 14,

### Western Reserve University, Dental College.

The annual commencement of the dental department of Western Reserve University was held Tuesday, May 17, 1898, in Association Hall.

Rev. Dr. Louis Albert Banks delivered the graduating address, entitled "The Romance of Labor." President Charles F. Thwing, of the University, conferred degrees upon the following candidates:

| | | |
|---|---|---|
| Fred Fayette Chapman. | Thomas Bertell Johnson. | Everett Eugene Quirk. |
| Foster Oran Dudgeon. | Edward Raymond Kelley. | John Babcock Reeves. |
| Charles Surfestus Draime. | Herman Clifford Kenyon. | Oliver William Renkert. |
| Calvin Riley Edson. | Percy Ellis Maddock. | Edward Burton Rogers. |
| Charles Drawn Elder. | Frank R. Mathews. | James Frank Rybak. |
| Isidor Englander. | Joseph Worrell McDill. | Park Eugene Sprague. |
| Albert Carl Fisher. | John Allen McGannon. | Robert Harrison Wallace. |
| William Woods Gillen. | Francis Joseph McGannon. | Leon Clark Vincent, Ph. B. |
| Penrose Thomas Haight. | Frank Edgar Noland. | |
| George Eells Hervey. | | |

### Pittsburg Dental College.

The second annual commencement exercises of the Pittsburg Dental College (Department of Western University of Pennsylvania) were held in the Duquesne Theater, Pittsburg, Pa., on Friday afternoon, April 1, 1898.

The faculty address was delivered by Dr. J. G. Templeton, dean of the dental department.

The number of matriculates for the session was one hundred and fifty-nine.

The degree of D.D.S. was conferred on the following graduates by Chancellor Holland:

| NAME. | RESIDENCE. | NAME. | RESIDENCE. |
|---|---|---|---|
| F H Bassett | West Virginia | F C Hum | Pennsylvania |
| N J Baxter | Pennsylvania | R S King | Pennsylvania |
| H J Brown | Pennsylvania | H T McCune | Pennsylvania |
| W F Erbey | Pennsylvania | R J Old | Pennsylvania |
| S P Earnest, M D | Pennsylvania | G W Otto | West Virginia |
| D W Flint | Pennsylvania | H B Reamer | Pennsylvania |
| F C Friesell | Pennsylvania | O M Sorber | Pennsylvania |
| A L Foote | Ohio | W R Watterson | Pennsylvania |
| C W Hunt | Pennsylvania | H W Wick | Pennsylvania |

Total 18.

### Meharry Medical College—Dental Department.

The twelfth annual commencement exercises of the Dental Department of Meharry Medical College (Central Tennessee College) were held in the Gospel Tabernacle, Nashville, Tenn., on Thursday, February 3, 1898.

The valedictory address was delivered by Thomas N. Harris, D.D.S.

The number of matriculates for the session was nineteen.

The degree of D.D.S. was conferred on the following graduates by Rev. J. Bradin, D.D.; president of the college: R. H. Cobb, Georgia; T. N. Harris, Alabama; G. W. Macker, South Carolina; J. E. Redmond, Tennessee. Total 4.

### Appointment and Qualifications of Delegates to the National Dental Association.

EDITOR DENTAL REGISTER:

*Dear Sir:*—In the next issue of your journal, will you please call attention to that section of the by-laws of the National Dental Association, which relates to the appointment and qualifications of delegates, which is as follows:

"ARTICLE III. SECTION 3. All delegate members shall be practitioners of dentistry. They shall be received only from permanently organized State dental societies. They shall be elected by ballot at some regular meeting of their society, and shall be members who have done meritorious work for the profession; but no person shall be received as a delegate who is in arrears for dues to this Association."

Also, "ARTICLE IV. SECTION 1. Each State society may send one for every ten of its active members, as delegates to this Association for one year, upon complying with the requirements of its Constitution; but no society shall be entitled to representation that does not adopt or substantially recognize the Code of Ethics of this Association."

The fact that the AMERICAN DENTAL ASSOCIATION received delegates from both local and State societies, renders it necessary to call attention to the fact that delegates to the NATIONAL DENTAL ASSOCIATION will be accepted only from the STATE societies, and that such delegates must be elected by ballot at a regular meeting of the Society.

By request of the President.

EMMA EAMES CHASE, Cor. Sec'y,
April 11, 1898. National Dental Association.

